# BMAL1 modulates senescence programming via AP-1

**DOI:** 10.18632/aging.205112

**Published:** 2023-10-10

**Authors:** Sarah K. Jachim, Jian Zhong, Tamas Ordog, Jeong-Heon Lee, Aditya V. Bhagwate, Nagaswaroop Kengunte Nagaraj, Jennifer J. Westendorf, João F. Passos, Aleksey V. Matveyenko, Nathan K. LeBrasseur

**Affiliations:** 1 Mayo Clinic Graduate School of Biomedical Sciences, Mayo Clinic, Rochester, MN 55905, USA; 2 Robert and Arlene Kogod Center on Aging, Mayo Clinic, Rochester, MN 55905, USA; 3 Epigenomics Development Laboratory, Epigenomics Program, Center for Individualized Medicine, Mayo Clinic, Rochester, MN 55905, USA; 4 Department of Physiology and Biomedical Engineering, Mayo Clinic, Rochester, MN 55905, USA; 5 Center for Cell Signaling in Gastroenterology, Mayo Clinic, Rochester, MN 55905, USA; 6 Department of Biomedical Statistics and Informatics, Mayo Clinic, Rochester, MN 55905, USA; 7 Department of Orthopedic Surgery, Mayo Clinic, Rochester, MN 55905, USA; 8 Department of Medicine, Division of Endocrinology, Metabolism, Diabetes, and Nutrition, Mayo Clinic, Rochester, MN 55905, USA; 9 Department of Physical Medicine and Rehabilitation, Mayo Clinic, Rochester, MN 55905, USA

**Keywords:** AP-1, circadian clock, cellular senescence, senolytic, transcription regulation

## Abstract

Cellular senescence and circadian dysregulation are biological hallmarks of aging. Whether they are coordinately regulated has not been thoroughly studied. We hypothesize that BMAL1, a pioneer transcription factor and master regulator of the molecular circadian clock, plays a role in the senescence program. Here, we demonstrate BMAL1 is significantly upregulated in senescent cells and has altered rhythmicity compared to non-senescent cells. Through BMAL1-ChIP-seq, we show that BMAL1 is uniquely localized to genomic motifs associated with AP-1 in senescent cells. Integration of BMAL1-ChIP-seq data with RNA-seq data revealed that BMAL1 presence at AP-1 motifs is associated with active transcription. Finally, we showed that BMAL1 contributes to AP-1 transcriptional control of key features of the senescence program, including altered regulation of cell survival pathways, and confers resistance to drug-induced apoptosis. Overall, these results highlight a previously unappreciated role of the core circadian clock component BMAL1 on the molecular phenotype of senescent cells.

## INTRODUCTION

Aging is associated with the emergence of multiple biological hallmarks, representative of diverse forms of molecular damage or alterations in fundamental cellular processes. Although distinct, there is clear evidence for their interactions. Studies of the interplay between such hallmarks are expected to advance the understanding of aging biology and, potentially, yield new opportunities for therapeutic intervention.

Two mechanisms of aging that markedly impact cellular health and function are cellular senescence and circadian dysregulation. Cellular senescence is a state of stable growth arrest which occurs in response to a variety of stressors, including DNA damage, telomere dysfunction, and oxidative stress [1]. Cell cycle arrest is initiated and maintained through the p53/p21 pathway and/or the p16^INK4a^ pathway, which prevent CDK4- and CDK6- mediated inactivation of retinoblastoma protein (Rb) to block cell cycle progression [2, 3]. Senescent cells accumulate with advancing age and commonly present with altered morphology [4], increased senescence-associated β-galactosidase (SA-β-Gal) activity [5], chromatin reorganization [6, 7], a robust secretome (referred to as the senescence-associated secretory phenotype (SASP)), and resistance to apoptosis [8–10].

Circadian rhythms are ~24-hour biological oscillations which evolved in response to predictable environmental changes [11]. Cells contain highly conserved molecular circuits comprised of transcriptional-translational feedback loops which drive ~24-hour oscillations of core circadian clock components. In the positive feedback loop BMAL1 and CLOCK heterodimerize and drive transcription of genes with E-box enhancer elements [12–14], including *Period* (*Per1, Per2, Per3*) and *Cryptochrome* (*Cry1, Cry2*) genes. PER and CRY heterodimers drive the negative feedback loop by translocating back into the nucleus and repressing their own transcription through interaction with BMAL1: CLOCK [11, 15]. BMAL1: CLOCK also drives transcription of retinoic acid-related orphan nuclear receptors REV-ERB (α, β) and ROR (α, β) which regulate transcription of *Bmal1* (the gene coding for BMAL1) via ROR response elements [16]. BMAL1 is the only non-redundant clock gene, indispensable for the generation of circadian rhythms [14] and is considered a master regulator of the circadian clock [17]. In addition, BMAL1 is a pioneer transcription factor (TF) that rhythmically controls chromatin accessibility and thus affects the activity of other TFs [18].

Experimental models with accelerated accumulation of senescent cells exhibit clinical manifestations of aging, including shortened lifespan, loss of fat and muscle, and organ dysfunction [19–21]. Correspondingly, genetic, and pharmacological elimination of senescent cells improves tissue structure and function in diverse models of age-related diseases [22]. The loss of BMAL1 in mice results in reduced lifespan and an acceleration of age-associated phenotypes, including sarcopenia, subcutaneous fat loss, and cataracts [23, 24]. Interestingly, while the loss of BMAL1 and other circadian genes is well documented in aging [25, 26], and while pharmacological interventions which restore circadian rhythmicity promote healthy aging [27, 28], few studies have investigated the relationship between senescence and circadian regulation. BMAL1 has been linked to many senescence-associated pathways, including redox regulation, nutrient metabolism, and genotoxic stress-response [29]. Moreover, recent studies have suggested BMAL1 rhythmicity is altered in senescent fibroblasts and exhibits a prolonged period and delayed phase [30, 31]. Taken together, this suggests that BMAL1 may influence senescence programming, however the mechanisms have not been explored.

Here, we examine the circadian clocks and molecular phenotypes of non-senescent and senescent cells, with and without BMAL1. Through integration and analysis of RNA-seq and BMAL1-ChIP-seq data we show that senescence alters the rhythmicity and binding of BMAL1, and that BMAL1 plays an important and previously unappreciated role in AP-1 regulation of senescence programming, including resistance to apoptosis.

## RESULTS

### Rhythmicity and abundance of core circadian clock components are altered in senescence

**Figure 1:**
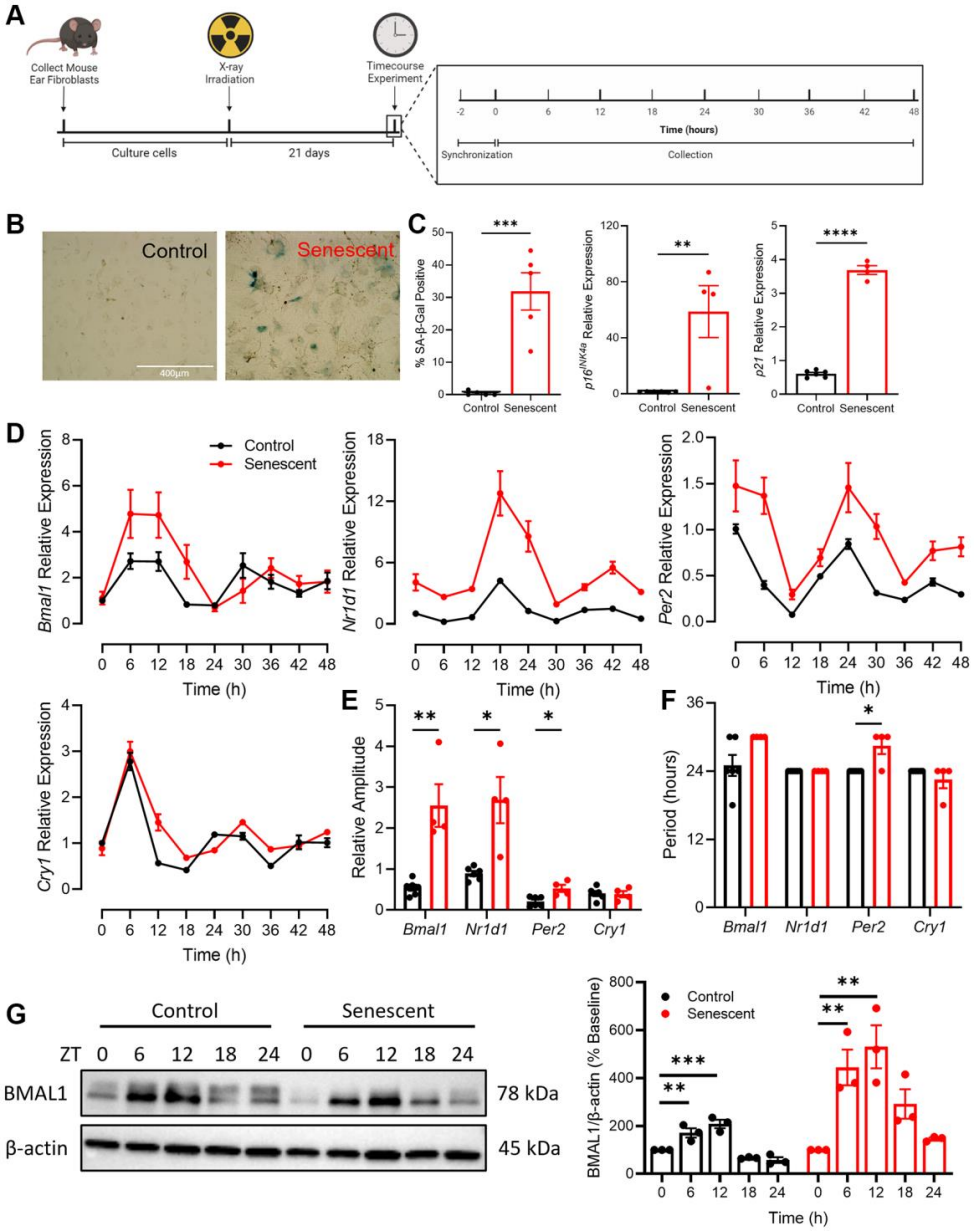
**Rhythmicity and abundance of core circadian clock components are altered in senescence.** (**A**) Experimental outline for collection and culturing of cells, senescence induction and time course experiment. (**B**, **C**) SA-β-gal staining and qPCR for CDKIs *p16^INK4a^* and *p21* in cells isolated from C57/BL6 mice. (**D**) Expression of core circadian clock genes *Bmal1, Nr1d1, Per2*, and *Cry1* in control and senescent cells. (**E**) Quantification of relative amplitude of *Bmal1, Nr1d1*, *Per2*, and *Cry1* in control and senescent cells. (**F**) Quantification of period of *Bmal1, Nr1d1*, *Per2*, and *Cry1* in control and senescent cells. (**G**) Representative western blot and quantification of BMAL1 in control and senescent cells, normalized to β-actin. Data is presented with mean +/− SEM; two-tailed unpaired Student’s *t*-test (**C**, **E**, **F**) and one-way ANOVA tests with Dunnett’s post hoc test for multiple comparisons (**G**) were used with significance indicated as ^*^P < 0.05, ^**^P < 0.01, ^***^P < 0.001, ^****^P < 0.0001. *n* = 4 replicates for senescent qPCR, *n* = 6 for control qPCR, *n* = 3 replicates for western blots. Scale bar in (**B**) is 400 μm.

To establish a model of senescence, mouse adult fibroblasts (MAFs) were isolated from the ears of C57BL/6 mice and were sham irradiated (control) or x-ray irradiated to induce senescence (Figure 1A). There was a significant increase in key markers of senescence, including altered cellular and nuclear morphology, senescence-associated β-galactosidase (SA-β-Gal) staining, and expression of CDKIs *p16^INK4a^* and *p21* in irradiated cells compared to the control cells (Figure 1B, 1C). To investigate the impact of senescence on circadian rhythmicity, we examined core circadian clock components in senescent and non-senescent contexts. Control and senescent cells were treated with 100 nM dexamethasone to synchronize their circadian clocks [32] and then collected every 6 hours for 48 hours (Figure 1A). Expression of core circadian clock genes *Bmal1*, *Nr1d1*, *Per2*, and *Cry1* were assessed by qPCR (Figure 1D) and the R package MetaCycle [33] was used to determine the rhythmicity of the time course data (Figure 1E, 1F). Compared to control cells, senescent cells had increased expression of *Bmal1*, *Nr1d1*, and *Per2* across the time course, with the most pronounced increases apparent at the peaks of each respective gene (Figure 1D). The amplitude, which is the height of peaks relative to baseline, of *Bmal1*, *Nr1d1*, and *Per2* expression was significantly increased in the context of senescence (Figure 1E). The period, which is the time elapsed during one complete circadian cycle, was increased by ~6 hours in *Bmal1*, and by ~4 hours in *Per2* expression (Figure 1F).

To determine if the differences in the *Bmal1* transcript in senescent cells were apparent in the abundance of BMAL1 protein, control and senescent cells were synchronized and collected every 6 hours for 24 hours (Figure 1G). In control cells BMAL1 protein abundance was highest 6- and 12-hours post synchronization (~2-fold increase) and decreased to slightly below baseline at 18- and 24-hours post synchronization, consistent with the pattern seen in the *Bmal1* transcript. In senescent cells, BMAL1 was also highest 6- and 12-hours post synchronization, however we noted a more robust (~5-fold) increase relative to the change observed in the control cells. Moreover, BMAL1 remained elevated at 18 hours in senescent cells before lowering at 24 hours to levels comparable to baseline. These alterations in BMAL1 protein are consistent with the increase in period and amplitude seen in the *Bmal1* transcript in senescent cells and corresponding augmented expression of key BMAL1 target genes (e.g., *Nr1d1* and *Per2*). Taken together, these data demonstrate an interaction between circadian and senescence programs as the abundance and rhythmicity of several core circadian clock components is significantly altered in the context of senescence.

### BMAL1 is localized at AP-1 binding sites in senescence

**Figure 2:**
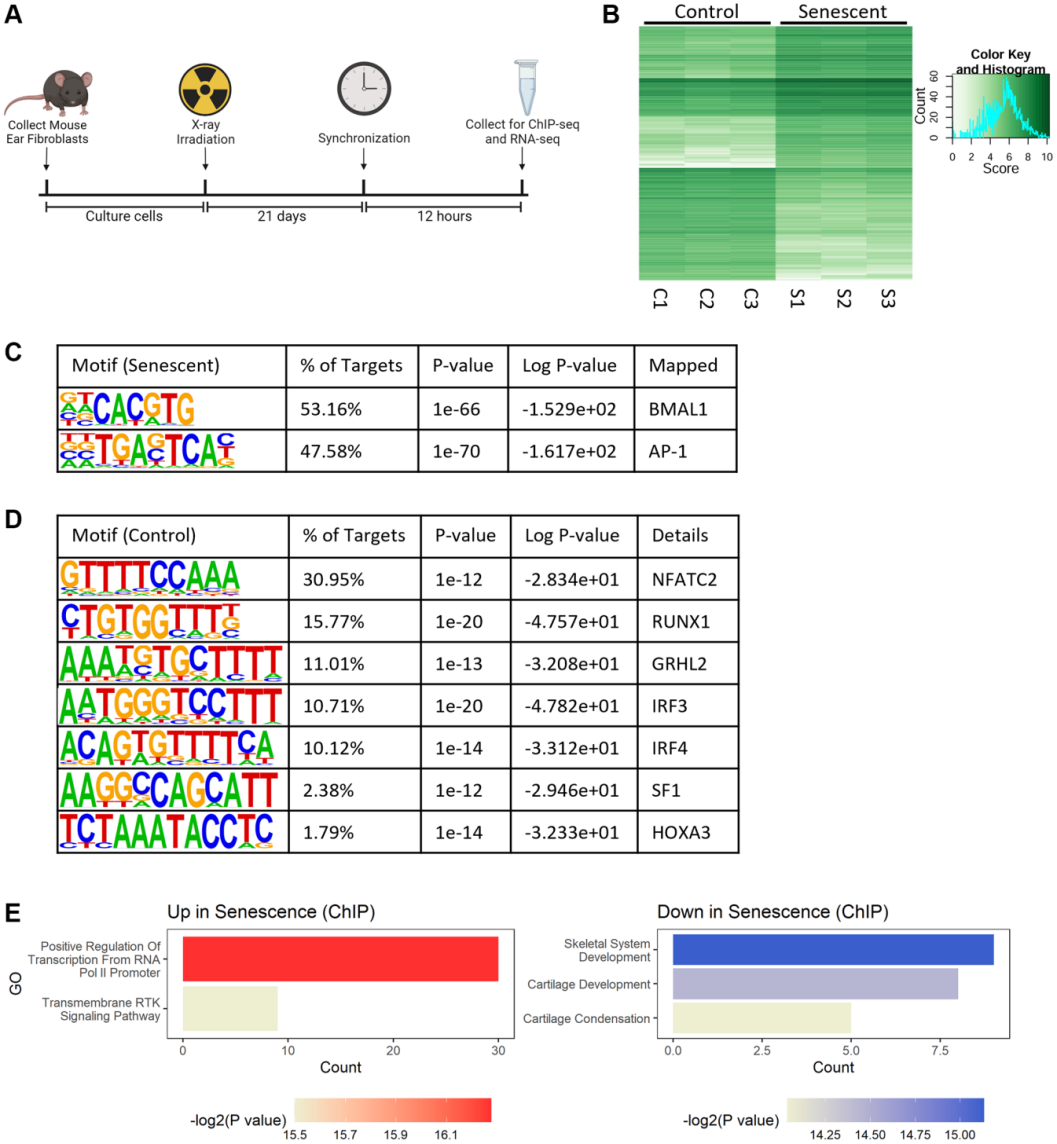
**BMAL1 is localized at AP-1 binding sites in senescence.** (**A**) Experimental outline for collection and culturing of cells, senescence induction and collection. (**B**) Differential binding between control and senescent cells identified by BMAL1-ChIP-seq. (**C**, **D**) Top motifs enriched in senescent cells (**C**) and control cells (**D**). (**E**) Gene ontology of pathways up- and downregulated in BMAL1-ChIP-seq targets in senescent cells. *n* = 3 replicates for BMAL1-ChIP-seq.

To study the global role of BMAL1 transcriptional activity in senescence, we synchronized control and senescent cells and collected them for BMAL1 chromatin immunoprecipitation with sequencing (BMAL1-ChIP-seq) 12 hours later (Figure 2A). This time was chosen to align with the highest abundance of BMAL1 protein (Figure 1G). BMAL1-ChIP-seq identified 605 differentially bound regions (logFC>|2|, *P* < 0.05) between control and senescent cells (Figure 2B). Motif analysis identified the two most abundant motifs enriched in senescent cells as the canonical E-box motif CACGTG, which is a known BMAL1 binding site [34] and, unexpectedly, the TGASTCA motif, which is a binding site of the AP-1 transcription factor [35] (Figure 2C). In contrast, there was only slight enrichment of several growth and proliferation associated motifs in control cells relative to senescent cells (Figure 2D). This suggests that while BMAL1 is bound to many genomic regions in healthy cells, this is lost in favor of more narrow binding in senescence with BMAL1 highly enriched at the E-box and AP-1 motifs. Consistent with this observation, gene ontology analysis of target genes identified by BMAL1-ChIP-seq in senescent cells showed an increase in BMAL1 binding to genes that plausibly influence the senescence program, including genes engaged in cell cycle arrest (Kdm6b, Smad3, Nr5a2) and MAPK signaling (Gdnf, Lif, Sall1), as well as a decrease in BMAL1 binding to genes associated with BMP pathway proliferative genes (Runx2, Bmpr1b, Col2a1, Col9a1, Col11a1). (Figure 2E).

Since BMAL1 is highly localized at AP-1 binding sites, we examined whether BMAL1 was directly interacting with AP-1 in senescence. We performed a BMAL1 co-immunoprecipitation on control and senescent cells collected 12 hours post-synchronization (Supplementary Figure 1A). Samples of the flow through, containing soluble proteins not bound to BMAL1, and samples of the elution, containing proteins bound to BMAL1, were collected and half of the samples were subjected to DNase digestion. In both control and senescent cells, c-Jun, a subunit of the AP-1 complex, was detected in samples of the flow through, and not in the elution, indicating that c-Jun protein was not directly bound to BMAL1. Additionally, no differences were detected between samples with or without DNase digestion, suggesting that DNA is not mediating potential binding behavior between BMAL1 and c-Jun. Another mechanism through which BMAL1 can affect cellular processes is by inducing rhythmicity in abundance of downstream targets. To test this, we performed a time course experiment for c-Jun in control and senescent cells post-synchronization (Supplementary Figure 1B) to determine if BMAL1 drives rhythmic behavior of c-Jun protein. We found no statistically significant change in c-Jun protein abundance at any timepoint post-synchronization compared to baseline abundance, in either experimental group (Supplementary Figure 1C), however we did note a significant increase (~2.3-fold) in c-Jun protein levels in senescent cells, in line with the importance of AP-1 in a senescence context (Supplementary Figure 1D). Together these data show that while BMAL1 is localized to AP-1 binding sites in senescence, BMAL1 is not directly binding to AP-1 or inducing rhythmic behavior.

### BMAL1 binding correlates with enriched gene expression in senescence

**Figure 3:**
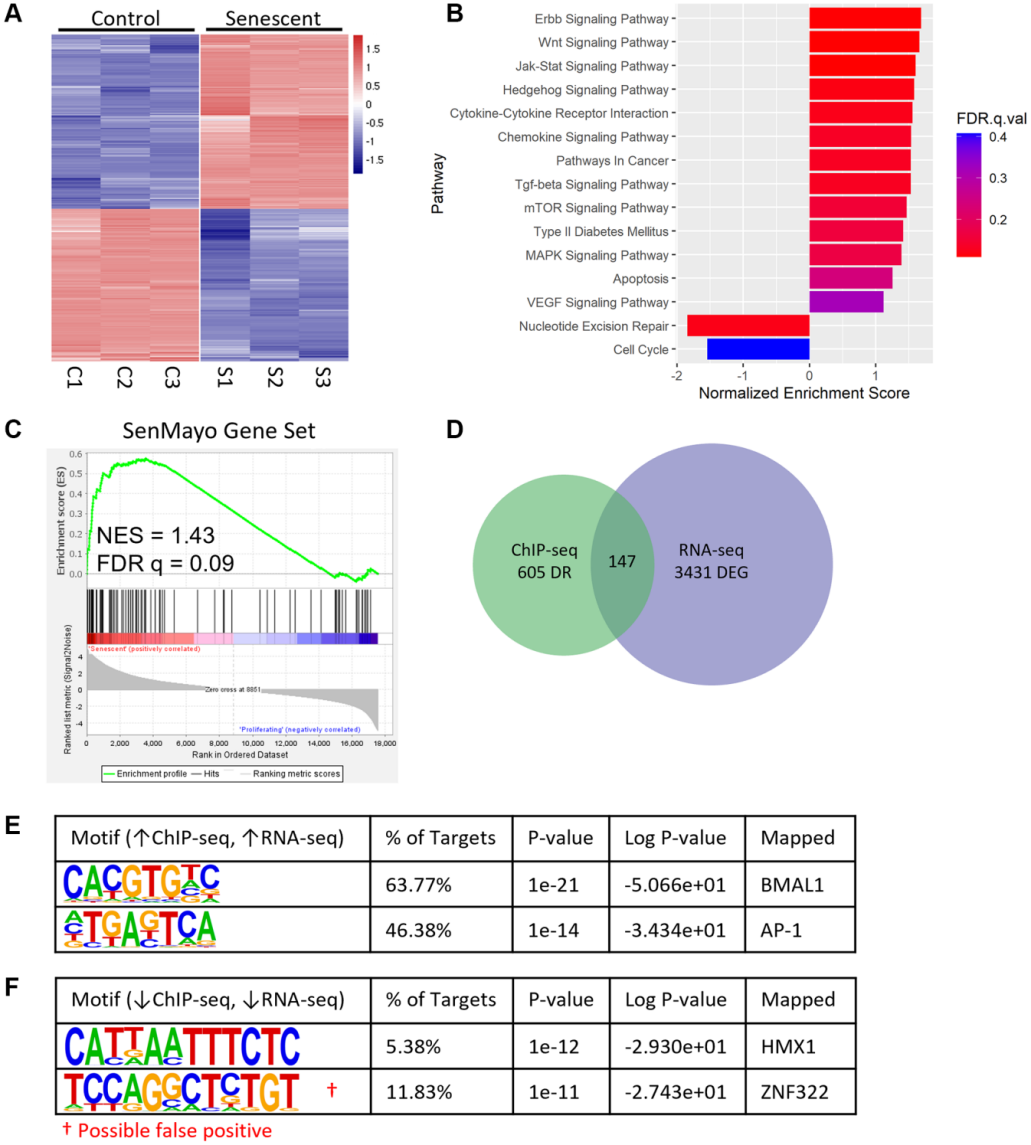
**BMAL1 binding correlates with enriched gene expression in senescence.** (**A**) Heatmap of DEGs from control and senescent RNA-seq. (**B**) Enriched pathways in senescent cells identified by KEGG, negative NES indicates pathways enriched in control cells. (**C**) GSEA enrichment plot of the SenMayo gene set in senescent cells. (**D**) Venn diagram of DEGs from RNA-seq and differentially bound regions from BMAL1-ChIP-seq. (**E**, **F**) Top motifs enriched in senescent cells with increased gene expression (**E**) and enriched in control cells with increased gene expression (**F**). *n* = 3 replicates for RNA-seq, *n* = 3 replicates for BMAL1-ChIP-seq. False positives are indicated with red^†^.

We next wanted to determine whether the high concentration of BMAL1 binding at AP-1 motifs drives differential gene expression in senescent cells. In addition to the control and senescent cells collected 12 hours post synchronization for BMAL1-ChIP-seq (Figure 2A) we also collected matched samples for RNA-seq. We identified 3,431 DEGs (fold change >2.0, *P* > 1 × 10^−5^) between control and senescent cells (Figure 3A). As expected, KEGG analysis showed significant enrichment of core properties of senescence, including Wnt, Jak-Stat, and chemokine signaling, and decreased enrichment of DNA repair and cell cycle pathways (Figure 3B). Moreover, gene set enrichment analysis (GSEA) showed significant enrichment of the SenMayo gene set, which identifies senescent cells across tissues and species with high fidelity [36], in senescent compared to control cells (Figure 3C). Integration of the BMAL1-ChIP-seq and RNA-seq datasets revealed 147 targets which were differentially bound in the BMAL1-ChIP-seq data and differentially expressed in the RNA-seq data (Figure 3D). Motif analysis of targets which were enriched in senescent cells in both datasets identified the CACGTG and TGASTCA motifs, mapping to BMAL1 and AP-1 respectively (Figure 3E), consistent with the binding pattern observed previously (Figure 2C). In contrast, only one target, HMX1, was enriched in both BMAL1-ChIP-seq and RNA-seq of control compared to senescent cells (Figure 3F). Moreover, there was no evidence of increased BMAL1 binding associated with decreased gene expression or decreased BMAL1 binding associated with increased gene expression (Supplementary Figure 2A, 2B). This demonstrates that the presence of BMAL1 at AP-1 binding sites in senescence is associated with increased gene expression.

### BMAL1 contributes to AP-1 mediated transcriptional control of the senescence program

The presence of BMAL1 at actively transcribed AP-1 target motifs suggested an important role of BMAL1 in AP-1 dependent transcriptional control of the senescence program, so we next investigated the senescence phenotype in cells deficient in BMAL1. We isolated cells from the ears of *Bmal1*−/− (KO) mice, which are completely arrhythmic and lack a functioning circadian clock [23], and induced senescence and characterized key senescence markers as described above (Figure 1A). We measured increases in SA-β-gal activity and expression of CDKIs *p16^INK4a^* and *p21* in a similar fashion to the WT cells (Supplementary Figure 3A, 3B), demonstrating that cells deficient in BMAL1 were able to initiate senescence programming and exhibit key markers of senescence. We also note the loss of the *Bmal1* transcript and lack of rhythmicity in other core clock components, as anticipated (Supplementary Figure 3C).

**Figure 4:**
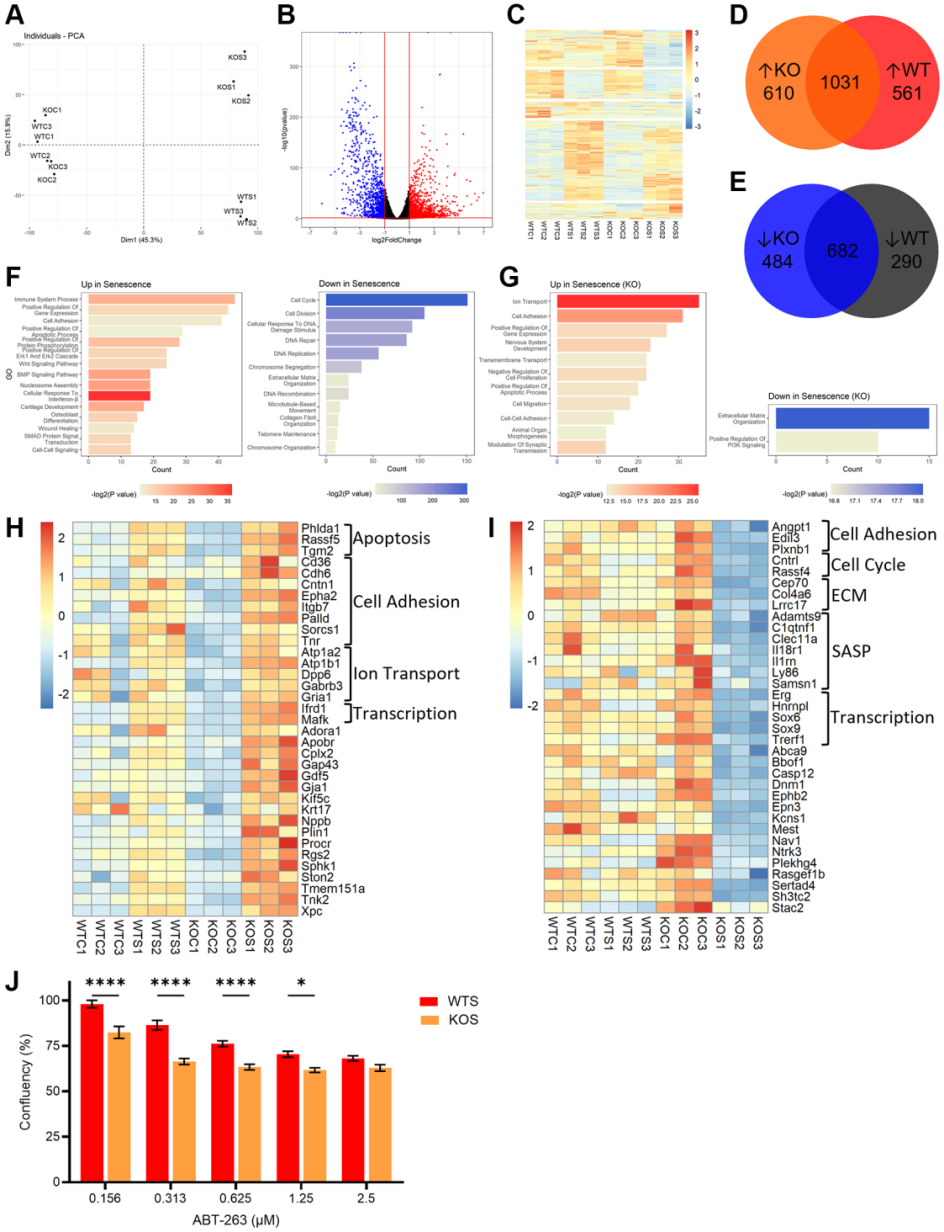
**BMAL1 contributes to AP-1 mediated transcriptional control of the senescence program and confers resistance to apoptosis.** (**A**) PCA plot of control and senescent WT and KO RNA-seq. (**B**, **C**) Volcano plot (**B**) and heatmap (**C**) of DEGs from WT and KO RNA-seq. (**D**, **E**) Venn diagrams of DEGs up- (**D**) and down- (**E**) regulated in WTS or KOS cells. (**F**, **G**) Gene ontology of pathways up- and downregulated in both WT and KO cells (**F**), and only in KO cells (**G**). (**H**, **I**) Heatmap of DEGs which are also AP-1 target genes upregulated (**H**) or downregulated (**I**) in KOS cells. (**J**) Confluence in senescent WT and KO cells after incubation with 0.156 μM, 0.313 μM, 0.625 μM, 1.25 μM and 2.5 μM ABT-263. Data is presented with mean +/− SEM; two-way ANOVA test with Sidak’s post hoc test for multiple comparisons were used (**J**) with significance indicated as ^*^P < 0.05, ^**^P < 0.01, ^***^P < 0.001, ^****^P < 0.0001; *n* = 3 replicates for RNA-seq. *n* = 16 replicate fields for WTS confluence and *n* = 12 replicate fields for KOS confluence.

To investigate global changes in senescence in the presence and absence of BMAL1, we performed RNA-seq on WT and BMAL1 KO control and senescent cells. PCA analysis showed separation between WT control (WTC) and KO control (KOC) compared to WT senescent (WTS) and KO senescent (KOS) cells, and separation between WTS and KOS cells (Figure 4A), highlighting overall differences in senescence programming between WT and KO senescent cells. Together, WT and KO cells exhibited 3,658 differentially expressed genes (DEGs) (fold change >2.0, *P* > 1 × 10^−5^) in senescent compared to non-senescent states (Figure 4B). These DEGs show distinct expression patterns between the four groups, including genes differentially expressed only in WT or KO senescent cells, and DEGs that are conserved in both (Figure 4C–4E). Gene ontology analysis showed expected changes in senescence-associated pathways, including an increase in immune system processes and Wnt signaling, and a decrease in cell cycle and DNA repair (Figure 4F). To focus on the impact of BMAL1 on the global senescence transcriptome, we further analyzed DEGs unique to KO senescent cells, and not differentially expressed in WT senescent cells. From these targets, we showed changes in several key pathways associated with the senescence program, including increased expression of inflammation response genes (Tlr4, Tnfrsf26, Csf1) and anti-apoptotic and pro-survival genes (Pou3f1, Pou4f1, Alox12), as well as decreased expression of BMP pathway proliferative genes (Sox9, Smoc1, Smoc2), and matrix metalloproteinases (Mmp17, Mmp23, Adamts9, Adamts15) (Figure 4G). Interestingly, no significant pathways or processes were identified from DEGs unique to WT senescent cells (data not shown).

We compared a list of AP-1 target genes [37] with our RNA-seq data in WT and BMAL1 KO senescent cells (Supplementary Figure 3D). We identified AP-1 target genes which were differentially expressed in KO senescent cells, but not WT senescent cells (Figure 4H, 4I). This analysis identified upregulation of genes associated with apoptosis and cell adhesion, downregulation of genes associated with the ECM and SASP, and interestingly both up- and downregulation of genes associated with transcriptional activity. This demonstrates that BMAL1 facilitates AP-1 transcriptional activity of senescence-associated targets, highlighting a meaningful role of BMAL1 in transcriptional control of the senescence program.

### BMAL1 confers resistance to apoptosis in senescent cells

Finally, given that senescent cells are resistant to apoptosis, we wanted to evaluate the significance of the BMAL1-dependent transcriptional changes, especially the changes in anti-apoptotic, pro-survival genes. We evaluated the dose response of WT and BMAL1 KO senescent cells to the senescent cell killing (“senolytic”) drug ABT-263 (navitoclax) which inhibits Bcl-2 family members and kills senescent fibroblasts in culture. We plated 5,000 senescent cells per replicate well and used a live cell monitoring system to measure confluence as a readout of senolytic activity. A greater reduction in confluence, indicative of greater senolytic activity, was evident in the KO senescent cells compared to WT senescent cells, at all except the highest dose (Figure 4J). This indicates that BMAL1 mediates the presence of pro-survival Bcl-2 family members and confers resistance to apoptosis in senescence, and the subsequent loss of BMAL1 sensitizes cells to ABT-263-induced cell death.

## DISCUSSION

In this study we discovered that senescence alters the amplitude and period of core circadian clock components, most notably BMAL1, at both a gene expression and protein level. The significance of this observation was confirmed by BMAL1-ChIP-seq, which demonstrated that BMAL1 binding in senescence uniquely and highly localizes to the motifs of AP-1, a pioneer TF previously identified as a key driver of the senescence transcriptome [38]. Correspondingly, integration of BMAL1-ChIP-seq data with transcriptomic data showed that high levels of BMAL1 in senescent cells drives expression of AP-1 target genes linked to DNA damage, growth arrest, and apoptosis resistance, i.e., core features of the senescence program. Moreover, the loss of BMAL1 alters the expression of AP-1 target genes and the transcriptional profile of senescent cells and confers resistance to apoptosis.

Recent work has shown an increased period length of BMAL1 in replicative senescent human lung fibroblasts [30] and in oxidative stress-induced human lung fibroblasts compared to controls [31]. In this study, we demonstrate similar changes in rhythmicity of BMAL1 in primary mouse fibroblasts (Figure 1). The consistency of our observation of increased BMAL1 period with previously reported findings further corroborates that alterations in molecular circadian clocks are a feature of cellular senescence. Given the importance of BMAL1 as a pioneer TF, we were interested in its binding pattern in a senescence context, since we observed changes in the abundance and rhythmicity of BMAL1, and especially considering the altered chromatin landscape in senescence [6, 7].

Recent work has shown that AP-1 is a pioneer TF and is crucial in priming the enhancer landscape in response to stress to ensure sequential activity of transcriptional events driving the senescence program [38]. AP-1 and its superfamily members were identified as pioneer TFs early in initiation of the senescence program, followed by later recruitment of settler TFs including NFY and the RELA subunit of NF-κB. Depletion of AP-1 and c-Jun in senescent cells resulted in a loss of the SASP, stemness, and anti-apoptotic senescence markers, highlighting the importance of this TF family in initiation and maintenance of the senescence program. Our BMAL1-ChIP-seq showed a significant enrichment of the TGASTCA motif, which maps to AP-1, indicating a role of BMAL1 in AP-1 regulation of the senescence program. By integrating BMAL1-ChIP-seq data with RNA-seq data, we determined that BMAL1 binding that is enriched in senescence is also associated with increased gene expression of BMAL1 and AP-1 target genes. By comparing the transcriptome of senescent BMAL1 KO cells to known AP-1 target genes, we demonstrate that BMAL1 is not merely present at sites of AP-1 transcriptional activity, BMAL1 is actively contributing to the AP-1 transcriptional regulation of senescence-associated genes.

We initially hypothesized that BMAL1 could be imparting circadian behavior on AP-1, however our BMAL1 co-immunoprecipitation showed no direct binding activity between BMAL1 and c-Jun, and our time course of c-Jun did not provide evidence of rhythmicity in control or senescent contexts. Together, these findings suggest BMAL1 is not directly regulating the abundance or rhythmicity of AP-1 protein, rather AP-1 transcriptional activity is substantially altered by the presence and activity of BMAL1, potentially through chromatin remodeling, histone modification, or recruitment of other activator or enhancer proteins. Further analysis of chromatin organization and epigenetic markers at AP-1 binding motifs in the presence or absence of BMAL1 will clarify the specific mechanism through which BMAL1 affects AP-1 transcriptional activity in senescence. This novel interaction between BMAL1 and AP-1 provides insight into the regulation of molecular pathways involved in senescence.

Given the importance of the molecular circadian clock in regulating stress responses, including oxidative stress and DNA repair pathways, we, and others, initially hypothesized that the loss of BMAL1 and subsequent loss of circadian rhythmicity would drive increased senescence induction. Initial characterization of *Bmal1−/−* mice demonstrated a reduced lifespan and an acceleration in age-associated phenotypes, including sarcopenia, subcutaneous fat loss, and cataracts [23] as well as increased SA-β-gal staining in multiple tissue types suggestive of an increased senescent cell burden [39]. As we and others have found, BMAL1 KO cells exhibit key senescence features, including growth arrest, increased SA-β-Gal activity and increased *p16* expression (Supplementary Figure 3) [39, 40]. When we further characterized the global transcriptome of senescent KO cells, we found significant changes in key senescence-associated pathways, implying that while loss of BMAL1 does not prevent or drive senescence, it does impact features of the senescence phenotype. One of the key areas of interest to us was the identification of anti-apoptotic factors as AP-1 targets mediated by BMAL1. Given the importance of senescence in health and aging, there is substantial interest in drugs which can overcome the apoptosis resistant nature of senescent cells and successfully reduce their numbers, such as the Bcl-2 family inhibitor ABT-263 (navitoclax) [9, 10]. We hypothesized that BMAL1 deficient cells would be susceptible to drugs which target these pathways, and indeed, we observe that senescent BMAL1 KO cells exhibit increased susceptibility to this senolytic agent, highlighting an important functional consequence of BMAL1 in driving key regulatory pathways of the senescence program.

## CONCLUSION

**Figure 5:**
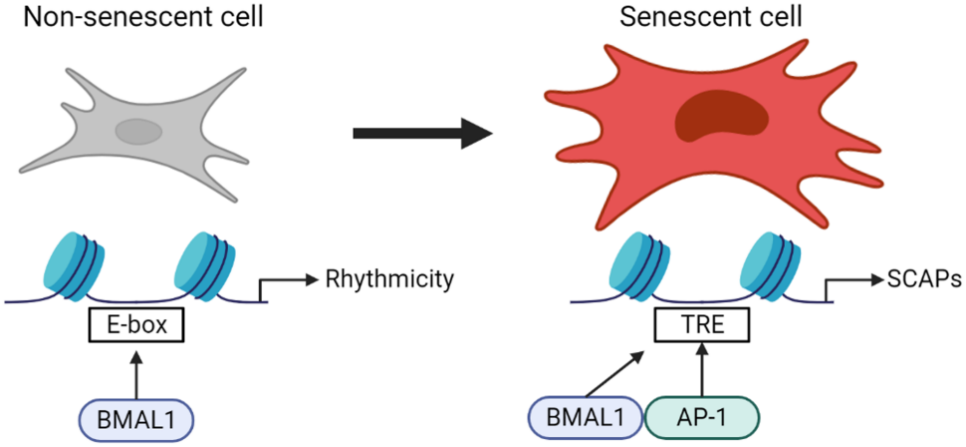
**Normal behavior of BMAL1 in driving transcription in non-senescent cells is lost in favor of narrow binding to AP-1 binding sites in senescence, and in a senescent context BMAL1 mediates expression of AP-1 target genes conferring resistance to apoptosis.** This highlights a previously unappreciated role of the core circadian clock component BMAL1 on the molecular phenotype of senescent cells.

Here, we propose a previously unappreciated role of the core circadian clock component BMAL1 in AP-1 transcriptional control of the senescence program. In this study we show that the normal behavior of BMAL1 in driving transcription in non-senescent cells is lost in favor of narrow binding to AP-1 binding sites in senescence, and in a senescent context BMAL1 mediates expression of AP-1 target genes conferring resistance to apoptosis. (Figure 5).

## METHODS

### Senescence-associated β-galactosidase (SA-β-Gal) staining

MAFs were fixed in phosphate-buffered 4% paraformaldehyde for 10 minutes at room temperature. MAFs were washed twice with PBS and incubated 16–18 hours in the SA–β-Gal staining solution (1 mg/mL X-Gal, 40 mM citric acid/sodium phosphate buffer pH 6.0, 5 mM potassium ferrocyanide, 5 mM potassium ferricyanide, 150 mM sodium chloride, and 2 mM magnesium chloride) on a shaker and in the dark at 37°C. MAFs were washed twice with PBS and stained with Hoechst dye for 10 minutes as a nuclear stain. Images were taken under bright field for SA–β-Gal staining and then the same field under blue fluorescence channel for nuclear staining (EVOS XL Core, EVOS 5000).

### RNA isolation, cDNA synthesis and qPCR

RNA was isolated according to manufacturer’s instructions using TRIzol reagent (Invitrogen). RNA concentration was assessed by Nanodrop 8000 (Thermo Fisher Scientific). cDNA was synthesized using M-MLV reverse transcriptase (Invitrogen) and real-time PCR was performed with PerfeCTa FastMix II (QuantaBio) and the Applied Biosystems StepOne Plus Real-Time PCR system (Applied Biosystems). Gene expression was analyzed by the ΔΔCT method and normalized to TATA-box binding protein as a reference gene. The primers used are listed in Supplementary Table 1.

### Co-Immunoprecipitation

Total protein extracts were prepared from fibroblasts in cell lysis buffer (Cell Signaling) with protease inhibitor cocktail (Sigma) and phenylmethylsulfonyl fluoride. Immunoprecipitation was performed using the Dynabeads™ Protein G Immunoprecipitation Kit (Thermo Fisher Scientific) according to manufacturer’s instructions. TURBO™ DNase (Thermo Fisher Scientific) was used at 6 U where indicated. 1/20 input volume and 1/5 elution volume were subjected to Western blotting (described below). Antibodies are listed in the Supporting Information.

### Western blotting

Total protein extracts were prepared from fibroblasts in cell lysis buffer (Cell Signaling) with protease inhibitor cocktail (Sigma) and phenylmethylsulfonyl fluoride. Protein concentration was determined using the DC Protein Assay (Bio-Rad). Equal amounts of protein (3 μg) were resolved by SDS–PAGE and transferred to a PVDF membrane (Bio-Rad). The membrane was blocked with 5% nonfat dry milk and then incubated with primary and secondary antibodies. Antibodies are listed in the Supplementary Information. Signal was developed using SuperSignal West Pico PLUS Chemiluminescent Substrate kit (Thermo Fisher Scientific) and imaged. (iBright™ FL1500 Imaging System, Thermo Fisher Scientific).

### Tagmentation-assisted fragmentation ChIP (TAF-ChIP)

ChIP-seq was performed following the published low input TAF-ChIP protocol [41]. Briefly, cells were cross-linked with 1% formaldehyde for 10 min, followed by quenching with 125 mM glycine for 5 min at room temperature. Fixed cells were washed once with TBS and stored at −80°C. The frozen cell pellets were resuspended in 500 mL of RIPA buffer (10 mM Tris-HCl, pH8.0, 140 mM NaCl, 0.1 mM EDTA, 1% Triton X-100, 0.1% SDS) and incubated on ice for 5 min. The lysates were sonicated for 4 cycles of 30 sec-on and 30 sec-off in Bioruptor pico (Diagenode) and centrifuged at 15,000 rpm for 10 min. The supernatant generated from 1 – 1.5 million fixed cells was mixed with 7.5 μL Protein A+G magnetic dynabeads (Millipore) conjugated with 1 μg of anti-BMAL1 antibody (Abcam) and incubated at 4°C overnight. The beads were washed once with 300 mL of RIPA buffer and twice with 300 mL of tagmentation buffer (20 mM Tris(hydroxymethyl) aminomethane pH 7.6, 10 mM MgCl_2_, 20% (vol/vol) dimethylformamide) using magnetic rack. The washed beads were suspended in 20 mL of tagmentation buffer containing 1 mL of Nextera DNA tagmentation enzyme (Illumina) and incubated at 37°C for 40 mins with constant shaking in a thermoblock at 500 rpm. And the beads were washed with RIPA buffer 2 times, RIPA buffer containing 250 mM NaCl 4 times, and TE buffer 2 times. Bound chromatins were eluted and reverse-crosslinked at 65°C overnight. DNAs were purified using Min-Elute PCR purification kit (Qiagen) after treatment of RNase A and proteinase K. All other details were followed as previously described (Zhong et al., 2017). The libraries were prepared from purified ChIP and input DNAs using NEBNext High-Fidelity PCR mix (NEB) with Nextera PCR primers with molecular indices (Nextera DNA Sample Preparation Kit) and sequenced to 51 base pairs from both ends using the Illumina HiSeq 4000 in the Mayo Clinic Medical Genomics Core. The ChIP enrichment was analyzed in BMAL1-positive and negative genomic loci by performing qPCR. The following primers were utilized; BMAL1-positive upstream of *Per2* gene (forward, ATAGTGGAAAACGTGACCGC; reverse, TGCAAATGAGGTGGCACTCC) and a background intergenic region (forward, GACAACCCTGGGGTAAGCTC; reverse, TCTGAGCGTCCACATCAGTG).

### RNA-sequencing

RNA-seq with WT and KO samples (Figure 4) was performed by Azenta Life Sciences. RNA samples were quantified using Qubit 2.0 Fluorometer (Life Technologies) and RNA integrity was checked using Agilent TapeStation 4200 (Agilent Technologies). RNA sequencing libraries were prepared using the NEBNext Ultra II RNA Library Prep for Illumina using manufacturer’s instructions (NEB). Briefly, mRNAs were initially enriched with Oligod(T) beads. Enriched mRNAs were fragmented for 15 minutes at 94°C. First strand and second strand cDNA were subsequently synthesized. cDNA fragments were end repaired and adenylated at 3’ends, and universal adapters were ligated to cDNA fragments, followed by index addition and library enrichment by PCR with limited cycles. The sequencing libraries were validated on the Agilent TapeStation (Agilent Technologies) and quantified by using Qubit 2.0 Fluorometer (Invitrogen) as well as by quantitative PCR (KAPA Biosystems).

The sequencing libraries were clustered on a lane of a HiSeq flowcell. After clustering, the flowcell was loaded on the Illumina instrument (4000 or equivalent) according to manufacturer’s instructions. The samples were sequenced using a 2 × 150bp Paired End (PE) configuration. Image analysis and base calling were conducted by the Control software. Raw sequence data (.bcl files) generated by the sequencer were converted into fastq files and de-multiplexed using Illumina’s bcl2fastq 2.17 software. One mismatch was allowed for index sequence identification.

RNA-seq that was matched with BMAL1-ChIP-seq (Figure 3, Supplementary Figure 2) was performed as follows. Total RNA concentration and quality were determined using Qubit fluorometry (Invitrogen) and the Agilent BioAnalyzer using the Total RNA Pico chip (Agilent). cDNA libraries were prepared using 200 ng of total RNA according to the manufacturer’s instructions for the TruSeq Stranded mRNA Sample Prep Kit (Illumina). The concentration and size distribution of the completed libraries were determined using an Agilent TapeStation D1000 and Qubit fluorometry. Libraries were sequenced at six samples per lane following the standard protocol for the Illumina NovaSeq™ 6000 and using the NovaSeq XP 2-Lane kit for individual lane loading. The flow cell was sequenced as 100 X 2 paired end reads using the NovaSeq SP sequencing kit and NovaSeq Control Software v1.7.5. Base-calling was performed using Illumina’s RTA version 3.4.4.

### ABT-263 time course

MAFs were plated in 96 well cell culture plates at a seeding density of 1,000 control cells per well and 5,000 senescent cells per well. Cells were treated with ABT-263 (APExBIO) at 0.156 μM, 0.313 μM, 0.625 μM, 1.25 μM, and 2.5 μM. Plates were incubated in an Incucyte SX5 live cell imaging system (Sartorius) for 62 hours at 37°C in atmospheric oxygen.

### Quantification and statistical analysis

#### Image analysis

For SA-β-Gal staining, nuclei and SA-β-Gal positive cells were quantified with ImageJ. Western blots were quantified using iBright Analysis Software (Thermo Fisher Scientific). ABT-263 time course images were quantified using Incucyte Base Analysis Software (Sartorius).

#### Statistical analysis

All statistical analyses were performed with GraphPad Prism v9.4.1 software. Two-tailed unpaired Student’s *t*-test were used for Figures 1C, 1E, 1F, Supplementary Figures 1D and 3B. One-way ANOVA tests with Dunnett’s post hoc test for multiple comparisons were used for Figure 1G, and Supplementary Figure 1C. Two-way ANOVA test with Sidak’s post hoc test for multiple comparisons was used for Figure 4J. Results with *p* < 0.05 were considered significant. Data are denoted as mean ± SEM with significance indicated as ^*^P < 0.05, ^**^P < 0.01, ^***^P < 0.001, ^****^P < 0.0001.

#### Processing of RNA-seq data

For the RNA-seq with WT and KO samples (Figure 4) that was performed by Azenta Life Sciences, sequence reads were trimmed using Trimmomatic v.0.36 [42], and then mapped to the Mus musculus GRCm38 reference genome available on ENSEMBL using the STAR aligner v.2.5.2b [43]. Unique gene hit counts were calculated by using feature Counts from the Subread package v.1.5.2 [44]. Only unique reads that fell within exon regions were counted. Unique gene hit counts were calculated by using featureCounts from the Subread package v.1.5.2. Using DESeq2 [45] a comparison of gene expression between the groups of samples was performed. The Wald test was used to generate *p*-values and log2 fold changes. Genes with an adjusted *p*-value < 0.05 and absolute log2 fold change > 1 were called as differentially expressed genes for each comparison. A gene ontology analysis was performed on the statistically significant set of genes by implementing the software GeneSCF v.1.1-p2 [46]. The mgi GO list was used to cluster the set of genes based on their biological processes and determine their statistical significance.

For RNA-seq that was matched with BMAL1-ChIP-seq (Figure 3, S2), RNA-Seq data were analyzed using the MAPRSeq pipeline v3.1.4 [47]. Paired-end sequencing reads were mapped to the mouse genome (mm10) using STAR aligner [43]. Gene and exon expression quantification were performed using the Subread package [44] to obtain both raw and normalized (RPKM – Reads Per Kilobase per Million mapped reads) reads. Following alignment, secondary analyses, and quality control, the edgeR package v3.36.0 [48] was used for obtaining differentially expressed genes (DEGs). Genes were considered differentially expressed at a statistical significance threshold of *p*-value < 0.05 and an absolute log2 fold change value of greater than 1.

#### Processing of ChIP-seq data

ChIP-Seq data were analyzed using the HiChIP pipeline [49]. Paired-end sequencing reads were mapped to the mouse genome (mm10) using Burrows-Wheeler Aligner v0.5.9 [50]. Post-mapping, additional filtering using in-house scripts was performed and only read pairs with one or both ends mapping uniquely to the genome were retained for downstream analysis. In addition, reads arising from PCR amplifications were discarded as duplicate reads using Picard MarkDuplicates v1.67 [51]. Candidate BMAL1 binding regions were identified using peaks detected by the software package “Model-Based Analysis of ChIP-Seq” [52]. Statistically significant peaks were selected for analysis using a false discovery rate (FDR) cutoff of ≤1%. Genes nearest to the peaks and the distance of peaks from transcription start sites (TSS) were annotated using the HOMER package. For visualization of peaks across samples and compatibility with the Integrative Genomics Viewer (IGV), normalized tag density profiles with a window size of 200bp and a step size of 20bp were generated using Bedtools v2.16.2 [53] and in-house Perl scripts. For comparison and identification of differential peaks across conditions, the software package DiffBind v2.14.0 [54] was used at statistical significance thresholds of *p*-value < 0.05 and an absolute log2 fold change value of greater than 2.

#### Rhythmicity analysis

Rhythmicity of gene expression was quantified with MetaCycle v1.2.0 [33].

### Resource availability

#### Lead contact

Further information and requests for resources and reagents should be directed to and will be fulfilled by the lead contact, Nathan LeBrasseur (LeBrasseur.Nathan@mayo.edu).

#### Materials availability

This study did not generate new unique reagents.

#### Data and code availability

1. The BMAL1-ChIP-seq data (GSE229009), Ctrl and Senescent WT RNA-seq (GSE229010), and Ctrl and Senescent WT and Bmal1−/− RNA-seq (GSE229011) in this study have been deposited to the National Center for Biotechnology Information Gene Expression Omnibus (Series GSE229023).
2. This paper does not report original code.
3. Any additional information required to reanalyze the data reported in this paper is available from the lead contact upon request.

### Experimental model and subject details

Primary mouse adult fibroblasts (MAFs) were isolated from the ears of adult female C57BL6/J mice and *Bmal1*−/− mice, with minimal modifications [55]. Notably, enzymatic digestion was performed with collagenase Type 2 (Worthington) at 800 U/mL in DMEM with 1% penicillin-streptavidin-glutamine and 2.5% HEPES buffer (Gibco) for 1 hour at 37°C. Cells and digested tissue were washed with HBSS and digested for an additional 20 minutes in 0.05% trypsin (Gibco) at 37°C. Isolated cells were then cultured in DMEM with 10% FBS and 1% penicillin-streptavidin-glutamine (Gibco) and provided fresh media every 3 days. MAFs were maintained at 3% oxygen and used in experiments between passage 3 and passage 8. IR-induced senescent MAFs were exposed to 20 gy radiation using an RS2000 X-Ray Inducer (RAD Source Technologies), then cultured for 21 additional days. MAFs were synchronized with 100 nM dexamethasone (Sigma) for 2 hours, prior to collection in TRIzol reagent (Invitrogen) for qPCR or in cell lysis buffer (Cell Signalling) for western blotting.

## Supplementary Materials

Supplementary Figures

Supplementary Table 1
