# Supplementary material for: BMAL1 modulates senescence programming via AP-1
Source: Aging (Albany NY). 2023 Oct 10;15(19):9984–10009. doi: 10.18632/aging.205112 (PMC10599731; doi:10.18632/aging.205112)
Supplement: Supplementary Figures [file aging-15-205112-s001.pdf]

SUPPLEMENTARY FIGURES

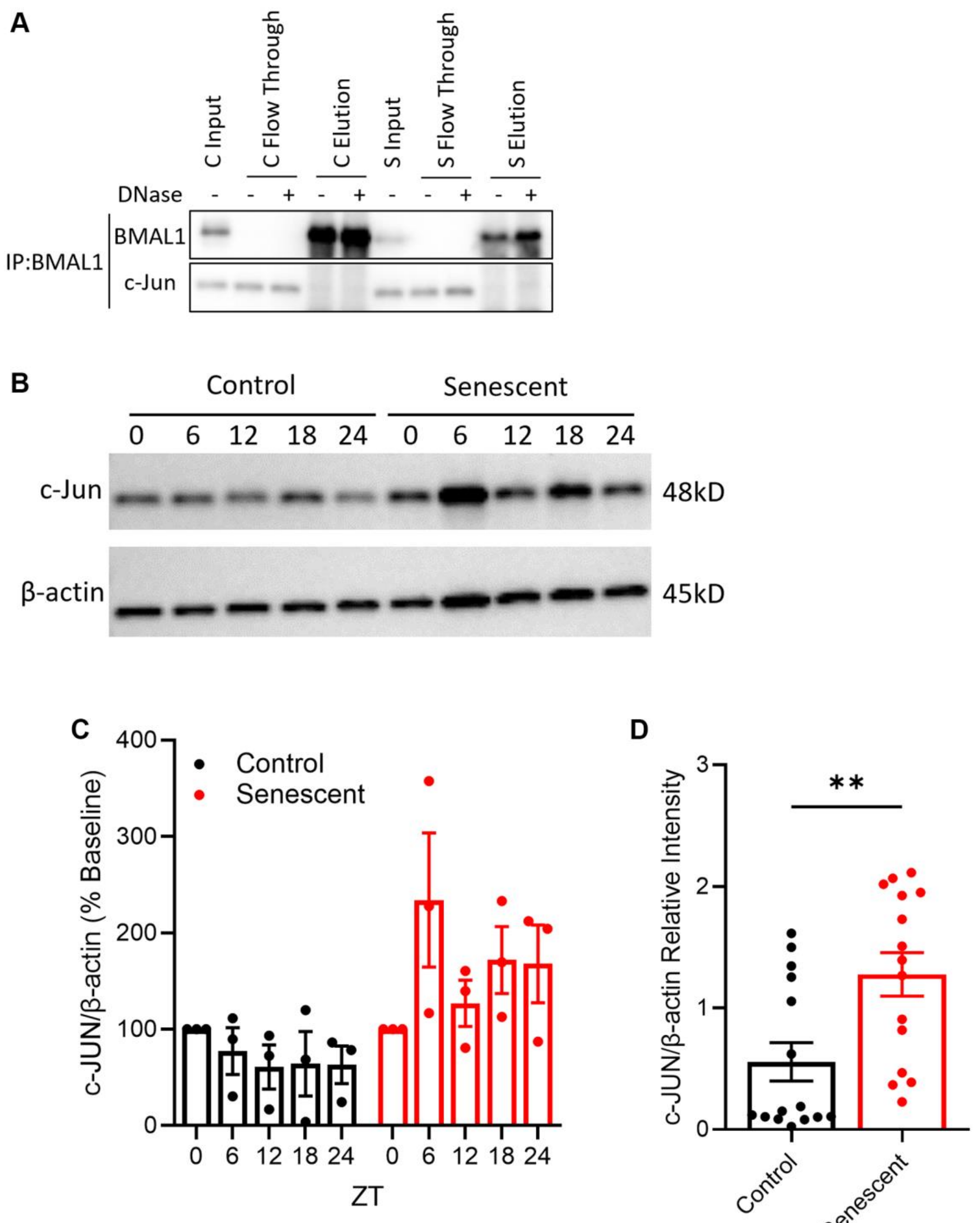

Supplementary Figure 1. BMAL1 is not directly bound and does not impart rhythmicity on c-Jun protein. (**A**) Representative BMAL1 co-immunoprecipitation in control and senescent WT cells, collected 12 hours post-synchronization, with or without DNase digestion. "Input" refers to 1/20 volume of input, "Flow Through" refers to 1/20 volume of flow through after binding of samples with BMAL1 antibody conjugated beads, "Elution" refers to 1/5 volume of final elution from beads. (**B**–**D**) Representative western blot (**B**) and quantification (**C**) of c-Jun in control and senescent cells, and relative intensity of all time points from each treatment group (**D**) normalized to β-actin. Data is presented with mean +/− SEM; one-way ANOVA tests with Dunnett's post hoc test for multiple comparisons (**C**) and two-tailed unpaired Student's *t*-test (**D**) were used with significance indicated as $^{*}P < 0.05$, $^{**}P < 0.01$, $^{***}P < 0.001$, $^{****}P < 0.0001$; $n = 2$ replicates for BMAL1 co-IP; $n = 3$ replicates for c-Jun WB.

A

Motif (↑ChIP-seq, ↓RNA-seq)	% of Targets	P-value	Log P-value	Mapped
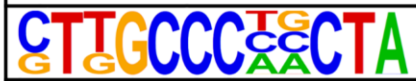 †	33.33%	1e-8	-1.913e+01	HIC2
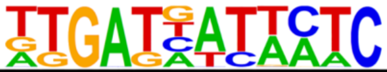 †	55.56%	1e-7	-1.667e+01	GATA

B

Motif (↓ChIP-seq, ↑RNA-seq)	% of Targets	P-value	Log P-value	Mapped
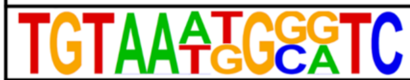 †	23.08%	1e-7	-1.798e+01	BSX
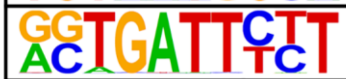 †	46.15%	1e-6	-1.603e+01	GFI1

† Possible false positive

Supplementary Figure 2. BMAL1 binding is not inversely correlated with transcription. Related to Figure 3. (**A**, **B**) Top motifs enriched in senescent cells with decreased gene expression (**A**) and enriched in control cells with decreased gene expression (**B**). *n* = 3 replicates for BMAL1-ChIP-seq. False positives indicated with red†.

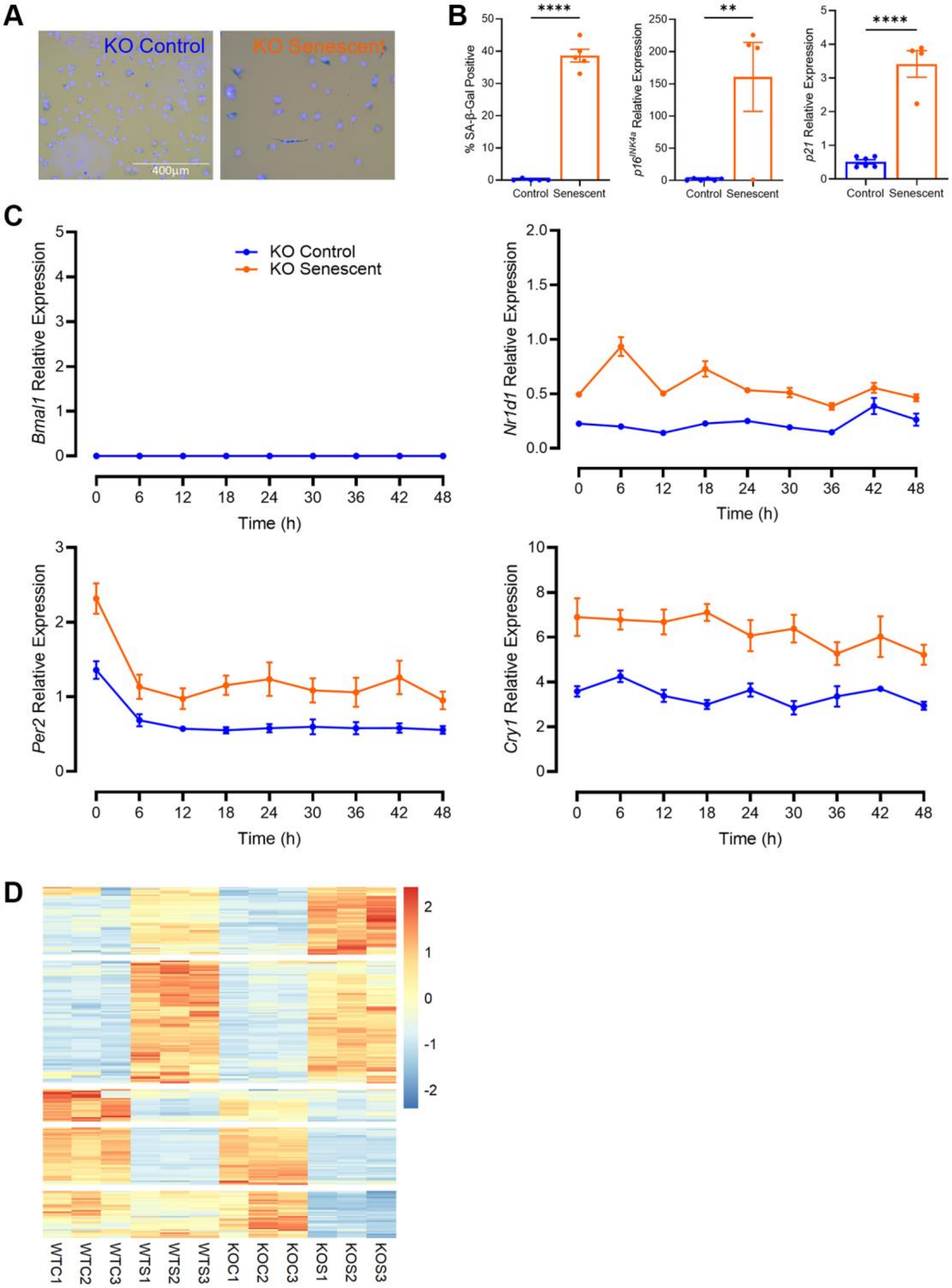

Supplementary Figure 3. BMAL1 deficient cells exhibit key senescence markers and are arrhythmic. (**A**, **B**) SA-β-gal staining and qPCR for CDKIs *p16*^{INK4a} and *p21* in cells isolated from *Bmal1 −/−* mice. (**C**) Expression of core circadian clock gene *Bmal1*, *Nr1d1*, *Per2*, and *Cry1* in control and senescent cells. (**D**) Heatmap of all DEGs which are also AP-1 target genes. Data is presented with mean +/− SEM; two-tailed unpaired Student's *t*-test (**B**) were used with significance indicated as $^{*}P < 0.05$, $^{**}P < 0.01$, $^{***}P < 0.001$, $^{****}P < 0.0001$. $n = 4$ replicates for senescent qPCR, $n = 6$ for control qPCR, $n = 3$ replicates for RNA-seq. Scale bar in (**A**) is 400 µm.
