# Supplementary material for: BMAL1 modulates senescence programming via AP-1
Source: Aging (Albany NY). 2023 Oct 10;15(19):9984–10009. doi: 10.18632/aging.205112 (PMC10599731; doi:10.18632/aging.205112)
Supplement: Supplementary Table 1 [file aging-15-205112-s002.pdf]

SUPPLEMENTARY TABLE

Supplementary Table 1. Key Resources Table.

Reagent or Resource	Source	Identifier
Primers		
Tbp	Integrated DNA Technologies	Cat# MM.PT.39a.22214839
Bmal1	Integrated DNA Technologies	Cat# Mm.PT.58.11121936
Cry1	Integrated DNA Technologies	Cat# Mm.PT58.6237142
Nr1d1	Integrated DNA Technologies	Cat# Mm.PT.58.17472803
p16INK4a	Integrated DNA Technologies	Cat# MM.PT.58.42804808
p21	Integrated DNA Technologies	Cat# MM.PT.58.5884610
Per2	Integrated DNA Technologies	Cat# Mm.PT58.5594166
Antibodies		
Rabbit anti-BMAL1	Cell Signalling	Cat# 14020 (1:1,000)
Rabbit anti-β-actin	Cell Signalling	Cat# 8457 (1:2,000)
Rabbit anti-c-Jun	Cell Signalling	Cat3 9165 (1:1,000)
Mouse anti-rabbit IgG-HRP	Santa Cruz	Cat# sc-2357 (1:20,000)
Rabbit anti-BMAL1	Abcam	Cat# ab3350
Chemicals, peptides, and recombinant proteins		
ABT-263 (navitoclax)	APExBIO	Cat# A3007
Cell lysis buffer	Cell Signalling	Cat# 9803
Collagenase Type 2	Worthington Biochemical	Cat# LS004176
Dexamethasone	Sigma	Cat# D1756
DMEM	Gibco	Cat# 11995-065
HEPES Buffer	Gibco	Cat# 15630-080
M-MLV Reverse Transcriptase	Invitrogen	Cat# 28025013
Penicillin-streptavidin-glutamine	Gibco	Cat# 10378-016
Protease inhibitor cocktail	Sigma	Cat# P8340
Protein A+G Magnetic Beads	Millipore	Cat# 16-663
PVDF membrane	Bio-Rad	Cat# 1620177
SuperSignal West Pico PLUS Chemiluminescent Substrate	Thermo Fisher Scientific	Cat# 34577
TRIzol reagent	Invitrogen	Cat# 15596026
TURBO™ DNase	Thermo Fisher Scientific	Cat# AM2238
0.05% Trypsin	Gibco	Cat# 25300054
Critical commercial assays		
DC protein assay	Bio-Rad	Cat# 5000116
Dynabeads™ Protein G Immunoprecipitation Kit	Thermo Fisher Scientific	Cat# 10007D
Min-Elute PCR purification kit	Qiagen	Cat# 28004
NEBNext Ultra II RNA Library Prep for Illumina	NEB	Cat# E7765
Nextera DNA Sample Preparation Kit	Illumina	Cat# FC-121-1030
NovaSeq 6000 SP Reagent Kit v1.5 (200 cycles)	Illumina	Cat# 20040719
NovaSeq XP 2-Lane Kit v1.5	Illumina	Cat# 20043130
TruSeq Stranded mRNA Sample Prep Kit	Illumina	Cat# 20020595

Deposited data		
BMAL1-ChIP-seq	GEO database	GEO: GSE229009
RNA-seq (Ctrl and Senescent WT)	GEO database	GEO: GSE229010
RNA-seq (Ctrl and Senescent WT and *Bmal1*−/−)	GEO database	GEO: GSE229011
Experimental models: Cell lines		
C57BL/6 WT MAFs	In house	NA
Bmal1−/− MAFs	In house	NA
Software and algorithms		
Prism v9.4.1	GraphPad	https://www.graphpad.com/scientific-software/prism/
ImageJ	ImageJ	https://imagej.nih.gov/ij/
iBright Analysis v5.1.0	Thermo Scientific	https://www.thermofisher.com/us/en/home/technical-resources/software-downloads/ibright-western-imager.html
Incucyte Base Analysis v2022A	Sartorius	https://www.sartorius.com/en/products/live-cell-imaging-analysis/live-cell-analysis-software/incucyte-base-software
GSEA v4.3.2	Broad Institute	https://www.gsea-msigdb.org/gsea/index.jsp
Factoextra v1.0.7	CRAN	https://cran.r-project.org/web/packages/factoextra/index.html
Pheatmap v1.0.12	CRAN	https://cran.r-project.org/web/packages/pheatmap/pheatmap.pdf
edgeR v3.36.0	[48]	
Trimmomatic v.0.36	[42]	
STAR aligner	[43]	
Subread v.1.5.2	[44]	
DESeq2	[45]	
GeneSCF v.1.1-p2	[46]	
MAPRSeq pipeline v3.1.4	[47]	
HiChIP pipeline	[49]	
Burrows-Wheeler Aligner v0.5.9	[50]	
Picard MarkDuplicates v1.67	[51]	
Model-Based Analysis of ChIP-seq	[52]	
Bedtools v2.16.2	[53]	
Diffbind v2.14.0	[54]	
MetaCycle v1.2.0	[33]	
